# Giant congenital nodular melanoma in a newborn: a case report and literature review

**DOI:** 10.1186/s12887-021-02590-7

**Published:** 2021-03-11

**Authors:** Jun Zhou, Meng-xing Lv, Ling Duan, Yu-cheng Xie, Zhi-xiang A, Hong-fang Wu, Yan Gao

**Affiliations:** 1 Department of Pathology, Kunming Children’s Hospital, 288 Qianxing Road, Yunnan 650028 Kunming, China; 2 Second People’s Hospital of Yunnan Province, 176 Qingnian Road, Yunnan 650034 Kunming, China

**Keywords:** Congenital, Melanoma, Pediatric, Proliferative nodules, Pathological presentation, Child, Case report

## Abstract

**Background:**

Malignant melanoma (MM) arises predominantly after adolescence and is uncommon in children. Congenital MM in newborns is even rarer with a dearth of published literature; as a consequence, there is no uniform standard for the pathogenesis and treatment for neonatal malignant melanoma. Herein we report a case of giant congenital nodular MM in a newborn, including its clinical, imaging, pathological and molecular pathological features. This case is the largest giant congenital primary nodular malignant melanoma in utero in neonates currently reported in China.

**Case presentation:**

A female neonatal patient was found to have a 2.97 cm× 1.82 cm×1.50 cm mass with a clear boundary at the right acromion in color Doppler ultrasound examination at 24 weeks of gestation. The mass increased to 3.0 cm×5.0 cm×9.0 cm at birth, and local ulceration was seen. MRI demonstrated that the mass was located on the right shoulder and underarm in a lobulated appearance, and surrounded the right scapula which was deformed. Clinical stage:IV(AJCC 8th Edition (2017)). α-Fetoprofein (AFP) by hematological examination: 1210ng/ml, NSE: 21.28ng/ml, LDH: 842U/L. The patient underwent surgical resection of the tumor, and was pathologically diagnosed as neonatal congenital malignant melanoma; immunohistochemistry (IHC): S-100 (+), HMB45 (+), Melan A (+), and Tyrosinase (+). Molecular pathological examination for BRAF V600E showed no mutations (Quantitative Real-time PCR, qPCR); And so were NRAS, C-kit (exons 9,11,13,14,17,18), and TERT (promoter locus, C228T and C250T) (Sanger sequencing). Non-surgical therapies were not carried out after the surgical resection of the tumor. After 6 months of follow-up, the child developed normally, and color Doppler ultrasound showed no obvious tumor growth or abnormality in the original tumor site.

**Conclusions:**

It is extremely rare to see giant congenital primary nodular MM in utero in neonates. The pathogenesis, treatment and prognosis of congenital MM need further research. The diagnosis mainly depends on histopathology and immunohistochemistry, and it needs to be differentiated from malignant lymphoma and primitive neuroectodermal tumor. The current treatment strategy for MM relies on the surgical excision of the mass. Research directed at molecular detection for genetic mutations would contribute to targeted therapy and better prognosis.

## Background

Malignant melanoma (MM) arises predominantly after adolescence, while the incidence in the prepubertal population is extremely low, and the incidence in people under the age of 20 is about 0.0005 %-0.0006 % [1], most of which are 10 or over the age of 10 years. The performance of patients aged 10–19 years is similar to that of melanoma in the adult population, while in people under the age of 10 years, the performance and prognosis of melanoma are significantly different from those of adults [2]. In comparison, the incidence of neonatal congenital MM is rare, especially for the congenital nodular melanoma with a very small number of reported cases. Neonatal congenital MM is characterized by lesions present on the skin at birth, however, little is known about the pathogenesis, treatment and prognosis. Herein a case of giant congenital primary nodular MM in utero in a newborn is described, with a brief review of literature, including clinical, imaging, pathological and molecular pathological features, which provides information for further research on this tumor.

## Case presentation

**Fig. 1:**
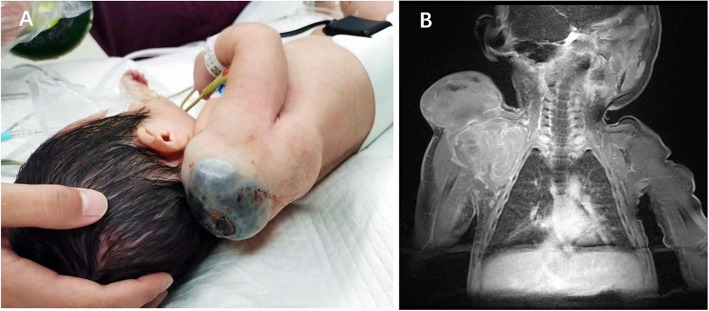
Giant nodular mass on the right shoulder with black skin surface and ulceration on the top of the skin, accompanied by a small amount of bright red blood exudation (**a**). MRI showing the localization of the tumor on the right shoulder and underarm with a lobulated appearance, surrounding the right scapula which was deformed (**b**)

**Fig. 2:**
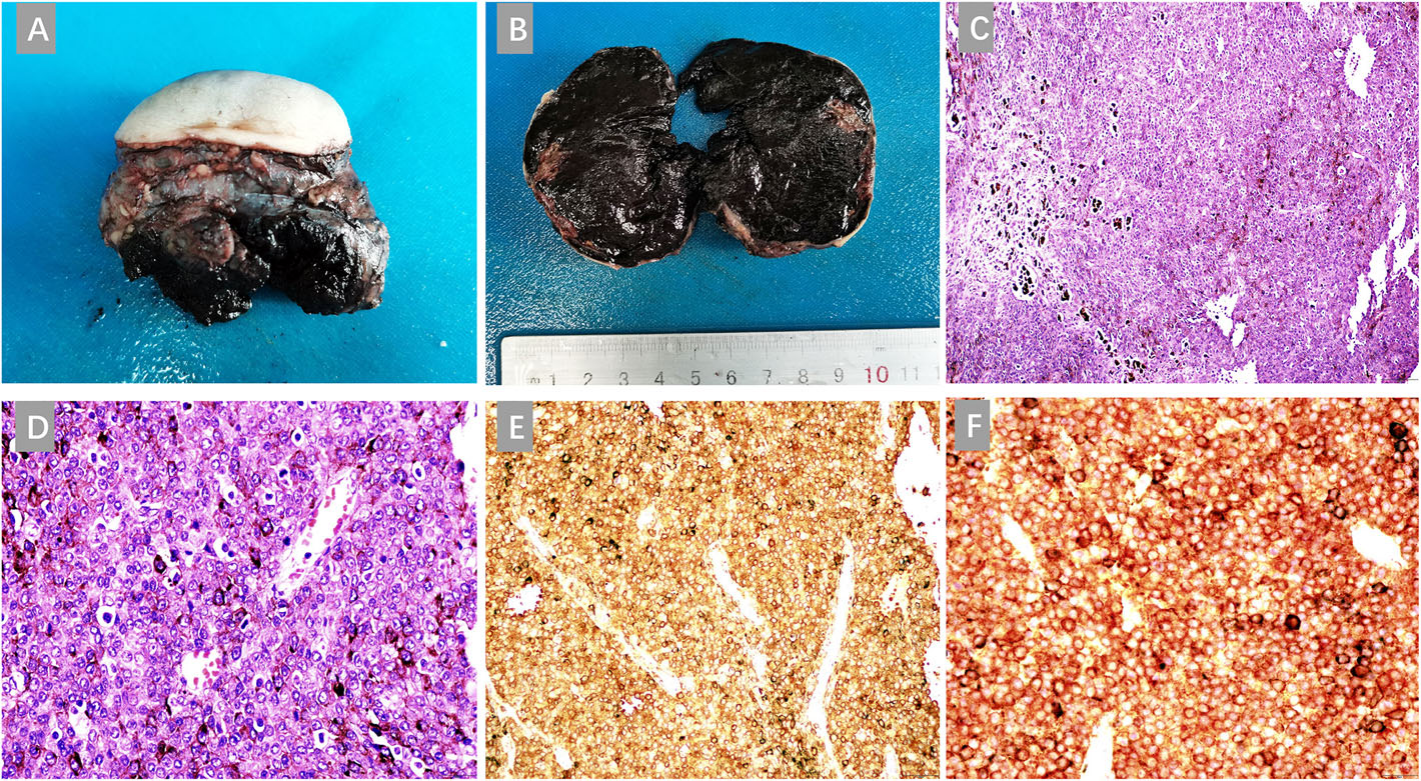
Surgically excised mass surface with skin and incomplete capsule, most of which were black (**a**). The section observed was solid and black, with necrosis present in certain parts (**b**). HE staining showed the distribution of tumor cells in the dermis in sheets or nests (magnification × 100) (**c**). HE staining showed round or oval tumor cell nuclei, with evident nucleoli and nuclear divisions were common. A large amount of melanin was seen in the cytoplasm, and local epithelium was absent (magnification × 400) (**d**). IHC showed Melan-A positive (magnification × 200) (**e**). IHC showed Tyrosinase positive (magnification ×400) (**f**)

A female baby (G1P1 38 + 6 weeks gestation) was born via cesarean section, weight 2.89 kg, no fetal distress, birth without asphyxia. The physical parameter were: body temperature 36.8 ℃, heart rate 152/min, and respiration 46/min. Serum total bilirubin was 123.7umol/L (3.4–17.7). The child was born with a huge nodular mass (3.0 cm×5.0 cm×9.0 cm) on her right shoulder. The skin on the surface of the tumor appeared black, with skin ulceration about 1 cm×1 cm on the top, accompanied by a small amount of bright red blood exudation (Fig. 1a). No melanin plaques were observed on the skin of other parts of the patient’s body. Magnetic Resonance Imaging (MRI) revealed that the tumor was located on the right shoulder and underarm in a lobulated appearance, and surrounded the right scapula which was deformed (The right scapula showed irregular spots and patchy bone destruction. The bone was discontinuous, with infiltrating tumor) (Fig. 1b), Clinical stage:IV(AJCC 8th Edition (2017)).The findings of AFP by hematological examination were 1210ng/ml (50-100000), NSE: 21.28ng/ml (0-16.3), LDH: 842U/L (67-394.1). The patient was found to have a mass of 2.97 cm×1.82 cm×1.5 cm on the right acromion in the color Doppler ultrasound examination at 24 weeks of pregnancy. Her parents and other family members denied a family history of any disease (hereditary, infectious, or similar diseases), mental illness, and malignant tumors. The infant underwent tumor resection and pathological examination. General examination of the tumor found the presence of a gray-red and dark-red skin mass measuring 9.0 cm×5.0 cm×4.0 cm with an incomplete capsule that was mostly black in color. A 2.0 cm×1.5 cm sized ulceration was seen on the surface of the mass (Fig. 2a). The section observed was solid and black, and necrosis was also present in certain parts (Fig. 2b). Histopathological examination revealed that the tumor cells in the dermis were distributed in sheets or nests (Fig. 2c), with round or oval nuclei, in which some nucleoli were evident and nuclear divisions were common. A large amount of melanin was seen in the cytoplasm, and local epithelium was absent. Infiltration and necrosis of neutrophils was observed, and the tumor invaded striated muscles,residual tumor was seen in the local margin (Fig. 2d). IHC staining showed the expression of S-100 (+), HMB45 (+), Melan A (+) (Fig. 2e), SOX10 (+) and Tyrosinase (+) (Fig. 2f), Ki67 (+, hot spots 15–20 %), CK (-), CD163(-). Molecular pathological examination for BRAF V600E showed no mutations (Quantitative Real-time PCR, qPCR). And so were NRAS, C-kit (exons 9,11,13,14,17,18), and TERT (promoter locus, C228T and C250T) (Sanger sequencing). Final pathological diagnosis verified the mass as neonatal congenital nodular MM, Breslow T4b, Clark V. Since the patient was a newborn, non-operative treatment such as chemotherapy and radiotherapy were not carried out after surgical resection of the tumor. After 6 months of follow-up, the child developed normally, and color Doppler ultrasound showed no obvious tumor growth or abnormality in the original tumor site.

## Discussion and conclusions

### Subtypes MM of the melanomas

 MM predominantly arises in adults but rarely in children, accounting for only 1–4 % of all melanoma cases [3], while congenital MMs in newborns are even rarer. The histology of MMs can be categorized into 4 types: superficial spreading melanoma (SSM), nodular melanomas (NM), lentigo malignant melanoma (LMM), and acral lentiginous melanomas (ALM). Of all the groups, SSM is the most prevalent regardless of age, while NM and spitzoid melanomas have been reported in children more often than adults. The LMM or ALM are prevalent in the adult population and rarely occur in pediatric age group. Based on patient’s age, pediatric melanoma can be classified into congenital, childhood (prepubescent; randomly classified as aged ≤ 10 years), or adolescent (postpubescent; arbitrarily classified as 11–19 years) sub-types [4]. These classifications are pertinent as the risk factors for these different age groups are very different. Melanoma in younger children tends to affect the head and neck region and extremities, while in older children, the trunk is the most commonly affected site [5].

### Pathways

 There are three possible pathways for the occurrence of congenital melanoma: (1) Trans-placental transmission - Melanoma accounts for 8 % of all tumor diagnoses during pregnancy making it one of the most common malignancies in young women. It is also the most frequent malignancy to undergo trans-placental transmission [6]. The prognosis is bleak for newborns suffering from extensive visceral metastases at birth, usually, death occurs within days or months. In these cases, the diagnosis of MM is made by visual and histopathological examination of the placenta [7]. (2) Congenital melanoma- This subtype usually originates from giant congenital melanocytic nevi (GCMN) and about 1–3 % of newborns suffer from congenital melanocytic nevus at birth, with the pigmented lesions contain actively proliferating melanocytes [8]. Melanoma in children is more likely to be caused by precursor lesions with about one-third of melanomas arising from a congenital melanocytic nevus or a dysplastic or changing nevus. GCMN generally refers to congenital nevi (usually larger than 20 cm in diameter) with an area larger than 2 % of the body’s surface area. The distribution of lesions is garment-like, with the trunk, head and neck being the commonly affected regions. MM can occur in any part of a giant congenital nevus, and occurs typically in trunk lesions. (3) Primary melanoma in utero it is derived from non-diseased skin [9], which is also an important source of congenital melanoma. The pathogenesis of primary melanoma in utero is unclear and it seems to be most common in the limbs.

### Molecular biology

 Diagnosis of pediatric melanoma using molecular techniques is an upcoming area. Currently, no mutations unique to pediatric MM have been found, and the biomarkers often overlap with the molecular changes of adult melanoma. Traditional cutaneous melanoma is usually associated with oncogenic mutations of BRAF and NRAS. BRAF mutations account for about 40 %-60 % of melanomas [10], which mainly arise in young patients. 80 % of BRAF mutations are V600E, causing a continuous activation of BRAF kinase as well as downstream signaling transmission pathways (such as MAPK and MEKERK), thereby promoting cell proliferation and tumor invasion and metastasis, leading to unrestricted cell growth [11]. BRAF is the main driving factor in pediatric melanomas, while NRAS is unique to GCMN associated melanomas [12]. Mutations in the NRAS gene help activate the mitogen-activated protein kinase (MAPK) signaling pathway, induce melanocyte production, and increase cell proliferation and survival. It is estimated that up to 25 % of CM carry NRAS genetic mutation, 80 % of which are Q61R, Q61K and Q61L point mutations > Particularly NRAS mutations occur in 21 % of superficial spreading melanoma, 31 % of nodules melanoma and 8 % of acral melanoma subtypes. It is believed that the pathogenesis of MM is mainly related to sunlight exposure and the Ultraviolet rays in sunlight damage the skin and induce DNA mutations [13]. Mutation of P16 or CDKN2A gene located on the short arm of chromosome 9 is the main reason for the high genetic susceptibility of MM [14]. In recent years, it has been found that there are many other mutations have been associated with the incidence of the melanocyte disease, such as KIT, HRAS, GNAQ, PTEN, TERT, NF1 and ARID2. With the application of whole genome sequencing (WGS) technology in the study of pediatric melanoma, multiple mutations (such as C-kit, TERT) are specific to pediatric melanoma [15]. However, to establish a clear connection, the molecular biology characteristics of pediatric MM need to be further research. The early onset for MM is a sign of genetic cancer susceptibility, but, unlike other common cancers that primarily arise in adulthood, melanomas can occur irrespective of age. Controversy exists in the literature as to whether childhood melanoma and adult melanoma differ biologically, and whether these two share any genetic or environmental risk factors [16]. Therefore, no correlation has been drawn between the age of melanoma diagnosis and corresponding risk assessment for the relatives.

### Classification and diagnosis

 According to the WHO (2018) classification of skin tumours [17], the pediatric melanoma is divided into four categories: De novo melanomas, Melanomas arising in congenital naevi, Spitz melanomas and Conventional adult-type melanomas. (1) De novo melanomas: These arise at birth or in prepubertal (and sometimes older) individuals. They commonly develop in the dermis, with an undifferentiated or so-called blast-like cytomorphology, and show small, medium, or large cell phenotypes. These melanomas often develop rapidly and require IHC and molecular evaluation for distinction from other poorly differentiated or undifferentiated malignant neoplasms. Some small cell melanomas in children may be indistinguishable from naevoid melanomas in adults. (2) Melanomas arising in congenital naevi: These usually arise in large or giant congenital naevi, at birth, in childhood or (sometimes) in older individuals. These melanomas often develop in the dermis or subcutis. Comparable melanomas are seen in adults. (3) Spitz melanomas: Occasionally, the pediatric and adult melanomas may exhibit features strongly suggesting a Spitz nevus, such as the epidermal cleft surrounding the dermal cell nest, large epithelioid cells and fasciculate spindle cells [18]. (4) Conventional adult-type melanomas: These usually have an epithelioid cell phenotype corresponding to the superficial spreading and nodular subtypes in adults. IHC staining and special staining are the mainstays in the diagnosis, include S-100, HMB45, Melan-A and Tyrosinase. Argentaffin staining also plays a part in determining whether the particles on pathological sections are melanin.

### Differential diagnosis

 It is necessary to distinguish nodular melanoma from benign nodular hyperplasia with melanocytic characteristics and other non-pigmented nodular tumors. (1) The proliferative nodules in CMN are atypical melanocytic proliferations in larger CMNs, which mainly arise at the neonatal stage, and are often presented as a dark brown to black plaque or nodule in a GCMN. Microscopically, it presents as nodules composed of round epithelioid or spindle cells against the background of dense and diffuse infiltration of small melanocytes. These nodules are often located in the upper-middle layer of the dermis, where intracellular heteromorphic nuclei and mitotic images can be observed. The distinguishing features of melanomas from naevi include their large size (i.e. >7 mm), ulceration, high mitotic rate (> 4 mitoses/mm^2^), mitoses in the lower third of the lesion, asymmetry, poorly demarcated lateral borders, lack of maturation, finely-divided melanin, and marked nuclear pleomorphism [19]. (2) Pediatric nodular melanoma, if nonpigmented needs to be distinguished from malignant lymphoma or PNET. MM small cell tumors are small in size, less cytoplasmic, diffusely distributed, and very similar to malignant lymphoma in morphology, however, a positive Leukocyte common antigen (LCA) for malignant lymphoma can distinguish the two; and Homer-Wright rosettes can be seen in PNET. CD99 and S-100 in IHC can be positive, but HMB45 is negative.

### Therapy

 Due to the rarity of pediatric melanoma the pediatric population has not been included in clinical trials for the treatment, therefore creating a lack of separate treatment standards. The Offenmueller’s study does not indicate the need for a different clinical approach in case of melanoma in children and adolescents. Thus, the present treatment strategies for pediatric patients have been derived from the adult population [20, 21]. Surgery remains the primary treatment for melanoma in children and adults. For pediatric patients with more advanced disease, biologic therapies are used much more commonly than chemotherapy or radiation therapy some of which are being discussed here [22]. In recent years, the management of local and systemic lesions has improved with the development of melanoma targeted drugs and immunotherapeutics. Since BRAF mutations are estimated to be present in about 50 % of melanoma patients, inhibitors targeted at these are also being used for treatment. Vemurafenib and Dabrafenib are both BRAF inhibitors approved by U.S. Food and Drug Administration for the treatment of melanoma and these agents specifically inhibit the intracellular signaling through mutated BRAF [23]. Other targets within the MAPK signal transduction pathway such as MEK1 and MEK2 are also being targeted by using specific inhibitors such as Trametinib for therapy in patients with BRAF mutated melanoma. Studies focusing on the role of c-kit and TERT related mutations in pediatric melanoma may open newer avenues leading to the development of targeted drugs. Another approach being pursued is the modulation of the immune system of the host to target melanoma. Immunotherapy drugs derived from monoclonal antibodies such as Ipilimumab suppress the cytotoxic T lymphocyte antigen-4 (CTLA-4) work by up-regulating the host immune system’s recognition of and response to tumor cells. Immunomodulatory agents such as Interleukin (IL)-2 work by activating the host immune system to identify and attack malignant cells. Specifically, interferon alfa-2b at high doses has shown promising results in children with melanoma with a tolerable risk-benefit profile [24]. Immunotherapy based on anti-PD1 antibodies such as Pembrolizumab or Nivolumab has the potential to increase the prognosis of patients with long-lasting effect [25]. A novel but less common approach using a variety of adjuvant chemotherapeutics such as cisplatin, dacarbazine, and vindesine has shown varying results on patients with advanced disease [26]. The relative rarity of pediatric melanoma has so far limited the evaluation of new therapies. Adequate clinical trials could not be conducted to establish separate treatment standards due to the insufficient number of cases. Nevertheless, significant progress has been made in the genomics analysis of pediatric melanoma in recent years, which will provide more accurate evidence for tumor treatment and prognosis in this group.

In conclusion, due to the rarity of giant congenital nodular MM in newborns and the lack of large-scale case studies, the pathogenesis, treatment and prognosis of congenital MM remain to be studied further. However, a recent surge in the pediatric MM cases and the continuous development of gene sequencing, has underlined the need for specific guidelines for the management of this population. Molecular biology studies directed at the identification of specific biomarkers will provide more evidence for the treatment and prognosis of MM in children.

## Data Availability

The datasets used and/or analyzed during the current study are available from the corresponding author on reasonable request.

## Abbreviations

AFP: α-Fetoprofein
MM: Malignant melanoma
IHC: Immunohistochemistry
SSM: Superficial spreading melanoma
NM: Nodular melanomas
LMM: Lentigo malignant melanoma
ALM: Acral lentiginous melanomas
GCMN: Giant congenital melanocytic nevi
WGS: Whole genome sequencing
LCA: Leukocyte common antigen
CTLA-4: Cytotoxic T lymphocyte antigen-4
